# Land use changes in Zhangjiakou from 2005 to 2025 and the importance of ecosystem services

**DOI:** 10.7717/peerj.12122

**Published:** 2021-09-23

**Authors:** Kaipeng Xu, Yanyan Chi, Rongfeng Ge, Xiahui Wang, Siyang Liu

**Affiliations:** Center of Eco-Environmental Zoning, Chinese Academy of Environmental Planning, Beijing, China

**Keywords:** Zhangjiakou, Land use, Ecosystem services, Water conservation, Sandstorm prevention, Biodiversity

## Abstract

Changes in local land use affect regional ecological services, development planning, and optimal use of space. We analyzed the effects of changes in land use from 2000 to 2025 on the spatial distribution of ecosystem services using CLUS-S modeling to evaluate ecosystem functions in Zhangjiakou, China. We found that the urban ecosystem area in Zhangjiakou increased and farmland decreased between 2000–2025. Water conservation was relatively high and was concentrated in the nature reserves of southern Zhangjiakou. Soil conservation was mainly distributed in eastern and southern counties. The results of the CLUE-S model showed that the relative operating characteristics of the six land use types were > 0.70, and the logistic regression equation was able to successfully explain the distribution pattern of the different types of land use.

## Introduction

Land use changes (LUCC) are a direct result of human impact on earth’s natural ecosystems (Bryan et al., 2018). Humans transform land through development, management, and utilization of natural resources. Recent ecological research has focused on the changing functions of ecosystems caused by transformations of land use (Schaefer et al., 2015). For that purpose, the land-use-transfer model is widely used to understand the process, mechanism, and ecological impacts of land use changes and to explore potential impacts on regional ecological security (Cao & Zhang, 2015). Future land use is often simulated with the cellular automation model, Markov model, neural network algorithm model, the system dynamics method, and the conversion of land use and its effects at small regional extension model (CLUE-S) (Rasmus et al., 2021).

The Chinese government has evaluated the effects of altered ecological conditions on economic, political, cultural, and social construction, and promoted and implemented a number of ecological civilization projects (Anne et al., 2018). The artificial surface expansion model is an effective tool used to address the degradation of ecosystem service functions. The model uses quantitative analysis of ecosystem service functions (Xiao, Hu & Xiao, 2017). It is important to explore the artificial land surface expansion model based on ecosystem services for regional development planning and land space optimization. The artificial land expansion model may lead land managers to modify land use plans to improve and conserve the function of ecosystem services (Cao et al., 2017).

Biodiversity and ecosystem services are intrinsically linked with and impact the well-being of humans (Resende et al., 2021). However, biodiversity and ecosystem services are increasingly threatened by human activities (Balasubramanian & Sangha, 2021). There is an effort to maintain the ecological services of natural ecosystems, however, natural disasters, heat island effects, and pollution resulting from human development limit ecological carrying capacity (Maldonado et al., 2018). The attempts by the Chinese government to engineer ecology may not be sufficient to provide the optimal benefit. From Zhangjiakou City as a case study for northern China, we can identify the impacts of land use changes on ecological service functions do provides scientific support for the planning for ecological security and improves the efficiency of ecological (Fang et al., 2018).

The construction of ecological security functions is a basic guarantee of regional security, and ecosystem services connect humans to their environment (Goldstein et al., 2012). Lack of awareness of the importance of ecosystem service functions, and extensive management of ecological resources resulted in a decreased capacity for ecosystems to provide ecosystem services (Leslie, 2018). Insufficient ecological interpretation of most ecosystem service functions directly affects the conservation and management of natural ecosystem services. However, expansion of ecological research has made it possible to increase the understanding of the importance of ecological services, and to realize that the conservation and promotion of ecosystem service functions is a prerequisite for sustainable development (Moreno-Mateos et al., 2017).

The eco-environmental effects of land use change can be determined by land use change structure analysis coupled with the natural resources impact assessment model (Mielnik et al., 2021). In our CLUE-S simulation study, the land use change process was simulated with high resolution land use data. The coupling of the CLUE-S model and other models was helpful to realize the decision-making goals of ecological protection and environmental management. We used the CLUE-S model to deal with ecosystem services and transactions. We comprehensively evaluated the invest model for coupling, and explored the ecological and environmental effects of changes in land use (Wang & Ren C. Zhou, 2021).

The CLUE-S model has been applied to the study of land use and cover change in different regions and at different scales (Huang, Huang & Liu, 2019). The CLUE-S model is a diverse model capable of addressing rapid economic growth areas, mining cities, urban fringe areas, inland arid areas, karst mountainous areas, watershed reservoir areas, villages and towns, and forest parks at regional and local scales. The CLUE-S model generally has a good applicability for the small and medium-sized land use and cover change processes (Jiang et al., 2017). The model different scenarios simulation analysis for environmental management. The use of this model assists in the prediction of future land use changes and may help to determine environmental effects of those changes. At present, the CLUE-S model is widely used to explore the impacts of land use changes on regional ecosystem services (Liu et al., 2017).

The Zhangjiakou area is located northwest of Beijing, forming its critical ecological barrier (Scown, Winkler & Nicholas, 2019). Water conservation, soil conservation, and sandstorm prevention contributed by Zhangjiakou’s ecosystem services directly affect the ecological security of Beijing. The impacts of land-use changes in Zhangjiakou on the planning and management of ecological services is not well-known. Quantifying the effects of land use changes on ecological service functions in Zhangjiakou will promote ecological security for the population of Beijing, sustainable management of ecosystem services, and reduction of human impact on the environment (Maas & Fabian, 2021). Here, we used a novel combination of methods, including the soil loss equation (USLE), the CLUE-S model, assessment of biodiversity maintenance, and a simulation map of ecosystem services to determine the effects of land use change on ecosystem service functions in Zhangjiakou. We aimed to: (1) analyze Zhangjiakou ecosystem composition and changes, (2) determine the spatial distribution of ecological service functions in Zhangjiakou, and (3) increase the awareness of the influence of artificial surface expansion on regional ecological service function.

## Materials and Methods

### Research area

Zhangjiakou is located approximately 160 kilometers northwest of downtown Beijing and can be divided into two distinctive landscape types: Bashang Plateau and Baxia Basin (Cai et al., 2018). The Bashang Plateau borders the southern edge of the Inner Mongolia Plateau, accounting for one-third of Zhangjiakou’s total land area, with an average altitude of about 1,400 m. The terrain is higher in the south and lower in the north with a hilly landscape (Han et al., 2016). The Baxia basin is elevated in the northwest and lower in the southeast, with rolling mountains and vertical and horizontal valleys. Multiple rivers pass through the basin, and fertile lands are widely distributed around rivers (Liu et al., 2018).

**Figure 1:**
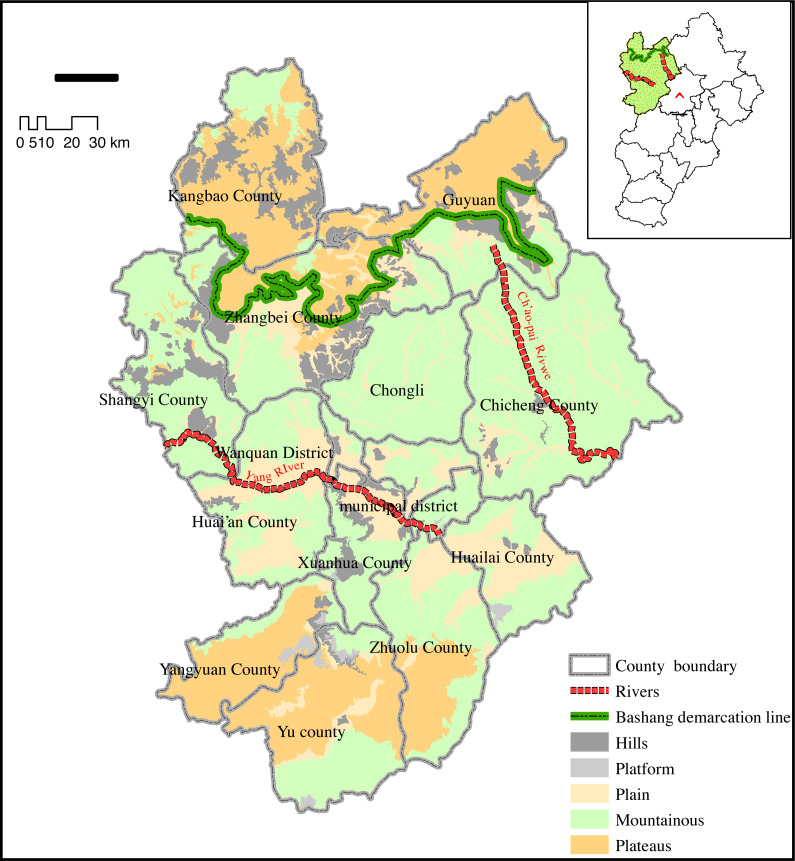
Diagram of Zhangjiakou Research Area. Zhangjiakou is a key area to maintain the ecological security of Beijing.

This area has a temperate continental monsoon climate with large temperature differences between night and day, hot and rainy seasons, long periods of cool weather, and few hot and humid days during the vegetation growing season (Lu et al., 2018). Valleys and basins are frequent in this area because the Sanggan River and Yanghe River run off the Baxia Basin, which is relatively low (Fig. 1).

Human activities have significantly degraded the ecosystem in this region and have created land desertification in some areas (Peng et al., 2016). We selected Zhangjiakou as the study area to evaluate the importance of ecological services. We used the CLUS-s model to study the impact of land use change, the importance of the spatial distribution pattern of ecological services, and the influence of future urban expansion on regional ecological service functions. Our results may serve as a reference for stakeholders in Zhangjiakou and contribute to the maintenance of regional and ecological security in Beijing (Wang et al., 2016).

### Data resources

We obtained land-use data from the 1:100,000 land use/land cover interpretation data provided by the Resource and Environment Science Data Center of the Chinese Academy of Sciences. Land-use classification included six categories: forest, grassland, wetland, cultivated land, artificial surface, and other landscapes. We used data from 2005, 2010, and 2015 to determine long-term land use changes in Zhangjiakou. The study area was selectively surveyed on the ground and results were used to correct and interpret satellite data for accuracy (Xiao & Xiao, 2019). Digital Elevation Model (DEM) data were obtained from the ASTER GDM V2 global digital elevation data of the geospatial data cloud. Data preprocessing was conducted to remove background values and obtain the DEM of Zhangjiakou City. We extracted the slope gradient and slope length using the spatial surface analysis function of ArcGIS (Xiao & Xiao, 2018). Administrative boundary, road, river, and residential area data were obtained from the National Basic Geographic Information Center (Zhao et al., 2018). Precipitation and temperature in and around the city were acquired from the meteorological information center of the National Meteorological Administration. Spatial distribution of meteorological stations was then used to interpolate annual rainfall, annual average temperature, and altitude. Statistical data, including population and administrative area, were based on the statistical yearbook of Zhangjiakou City from 2005-2015.

### CLUE-S model

CLUE-S model was developed and improved upon by Verburg of Wageningen Agricultural University in the Netherlands (Jiang et al., 2017). This model is based on the clue model, which was used to simulate small-scale land use changes. CLUE-S model considers correlation and competition among land use types. It can simulate land use patterns of different land use types in the same period. It is a dynamic and spatially dominant land use-cover change (LUCC) model.

CLUE-S model consists of two parts: the non-spatial and the spatial module (Zhang et al., 2013). The non-spatial module can calculate the total demand for each land use type. Land use demand is calculated as the prediction of land use change scenarios. This can be achieved by coupling a variety of other models, including the Markov chain and the economic model (Tran, 2021). The spatial module of the CLUE-S model is the core part of the model. The data in the spatial module are in the form of a grid that can allocate the overall land use demand. It can be transformed into grid-based land use patterns at different locations. Competition between various land types, conversion rules of land use, and the spatial distribution of land use demand in each simulation year can be decided based on the probability of land use types (Dey et al., 2021).

We used logistic regression for spatial analysis in the CLUE-S model; this is a commonly-used method in land-use change analysis (Johnson & Mueller, 2021). The regression equation was established in SPSS. Land use type was a dependent variable, and driving factors were independent variables. The stepwise regression method was also used to screen for significant factors affecting land use type, and non-significant factors were eliminated. The CLUE-S model was dual tested. First, the ROC coefficient i of logistic regression was tested, in which *i* > 0.7 met the requirements of probability distribution. Second, the kappa coefficient was tested for kappa ≥ 0.75 which indicates a higher prediction accuracy of CLUE-S model (Desta et al., 2021).

Simulation with the CLUE-S model involved four steps. In the first step, land use demand is estimated; land use demand refers to the demand for each land use type in a simulation period. Land use demand in this study was concerned with ecological protection. Human development was strictly restricted within the area of ecological protection. The change rate of cultivated land, grassland, water area, and unused land was practically zero (Wang, Zu & Wang, 2020). The growth rate of forest land may accelerate, while the growth rate of urban, industrial, and mining areas will decrease to adjust the demand of each land use type every year. The second step involved land use transfer flexibility and transfer rules. The CLUE-S model allows researchers to set the stability of different land use types according to historical changes in the land use system. Elas of 1 for land types means that no conversion to other land use types; Elas of 0 means land type will be easily converted, and a value between 0 and 1 indicates some conversion. It is determined by debugging in the simulation process. The third step was logistic regression analysis (Liu & Austin, 2021). Logistic stepwise regression was used to analyze the relationship between spatial distribution and driving factors of land use types. The fourth step involved a dynamic allocation of space and was mainly based on the initial land use type distribution status map, land use spatial distribution probability suitability map, driving factors, logistic regression equation, and conversion rules. The spatial distribution of land use demand is mainly based on the total probability (Martinez-Casasnovas, Ramos & Poesen, 2004).

### Assessment of functional importance of ecological services

We selected four types of ecological services including water conservation, water and soil conservation, sandstorm prevention, and biodiversity maintenance to evaluate the total ecosystem service function. We established comprehensive indicators of ecological services to quantify the relative importance of each grid unit to the maintenance of regional ecological security; indicators were based on the guidelines for delimiting the red line of ecological protection, and the accuracy of evaluation (Byakagaba & Muhiirwe, 2017). Each grid was sorted by the value of providing a specific service. The importance of a service function was assigned to one of three levels: extremely important, moderately important, and general. The levels were based on the maximum value, which determines the importance of each service and results in a comprehensive index of the importance of ecological services (Abdo Valeri et al., 2017).

#### Assessment of importance of water conservation

Water conservation was calculated using the following water balance equation (Gandhi & Sundarapandian, 2017):

\begin{eqnarray*}TQ=\sum _{i=1}^{j}({P}_{1}-{R}_{i}-E{T}_{1})\times {A}_{i} \end{eqnarray*}

where TQ is the total water conservation amount (m^3^), P _i_ is rainfall (mm), R _i_ is surface runoff (mm), ET _i_ is evapotranspiration (mm), A _i_ is class I ecosystem area (km^2^), i is class I ecosystem type in the study area, and j is the number of ecosystem types in the study area. Six ecosystem types were delineated in the study area, including forest land, grassland, wetland, and artificial surface.

#### Assessment of importance of water and soil conservation

The difference between potential and actual soil erosion was used to evaluate soil and water conservation function of an ecosystem. We used the modified equation of water loss erosion and soil as follows:

\begin{eqnarray*}{A}_{c}={A}_{p}-{A}_{r}=R\times K\times L\times S\times (1-C) \end{eqnarray*}

where *A*_*c*_ is soil and water conservation (T/hm^2^a); *A*_*P*_ is potential soil erosion, *A*_*R*_ is actual soil erosion, *R* is rainfall erosivity factor (MJ mm/hm^2^ha), *K* is soil erodibility factor (thm^2^h / hm^2^mjmm), *L* and *S* are terrain factors, *L* is slope length factor, *S* is slope factor, and *C* is vegetation cover factor.

#### Assessment of the importance of sandstorm prevention

We calculated the sandstorm prevention capacity of an ecosystem using the following formula (Jeong et al., 2017):

\begin{eqnarray*}{S}_{\mathrm{WS}}=NP{P}_{mean}\times K\times {F}_{q}\times D \end{eqnarray*}

\begin{eqnarray*}{F}_{q}= \frac{1}{100} \sum _{1=1}^{12}{u}^{3}\{ \frac{ET{P}_{1}-{P}_{1}}{ET{P}_{1}} \} \times d \end{eqnarray*}

\begin{eqnarray*}ET{P}_{1}=0.19(20+{T}_{1})^{2}\times (1-ri)u2=u(Z2~Z1)^{1/7}D=1/cos(\theta ) \end{eqnarray*}

where S _WS_ is the capacity index of sandstorm prevention service, *NPP*_*mean*_ is the average net primary productivity, K is the soil erosion factor, FQ is the average climate erosion, u is the monthly average wind speed at 2 m above ground, U1 and U2 are wind speeds at Z1 and Z2 heights, respectively, ETP _I_ is the monthly potential evaporation (mm), P _I_ is the monthly precipitation (mm), D is the number of days in a month, T _i_ is the average monthly temperature, r _i_ is the average monthly relative humidity (%), D is the surface roughness factor, and *θ* is the slope (radian).

#### Assessment of biodiversity maintenance

The maintenance of biodiversity is closely related to the distribution of rare, endangered, and endemic animals and plants. Species with first and second level protected status and other significant or flag species with important protection value were used to establish the biodiversity protection function of an ecosystem. Species distribution database was established by collecting animal and plant diversity data in the research area (Londe & De Sousa, 2017).

A species distribution model was used to quantify species dependence on the environment to predict the probability of a species presence in any area; this was done based on the attributes of the environment associated with the distribution of key species and their importance.

## Results

### Ecosystem types and their conversion in Zhangjiakou

Zhangjiakou City experienced a rapid increase in artificial surface area between 2000 and 2015, growing from 235.9 × 100 to 250.6 × 100 ha. The percentage of wetland increased during that time to 12.46%. The decline of farmland ecosystem was also evident, decreasing from 72.44 to 70.63%. The amount of transfer between ecosystem types included 13.9 × 100 ha of farmland to artificial surfaces (Table 1).

**Table 1:** Transfer between ecosystem types in Zhangjiakou (2000–2015).

	2015							
	Use Types	Wood Land	Grass Land	Wet Land	Cultivated Land	Artificial Surface	Others	Total Unit:km^2^
2000	Wood Land	10024.1	202.5	0.8	115.6	2.9	1.2	10369.2
	Grass Land	139.0	9702.0	15.7	366.5	16.9	9.9	10258.4
	Wet Land	1.0	31.6	304.2	48.2	1.4	17.3	404.3
	Cultivated Land	112.3	231.3	42.0	13462.7	51.4	5.0	13910.3
	Artificial Surface	29.2	129.4	0.9	369.9	973.3	2.1	1505.1
	Others	1.0	11.4	4.4	10.3	0.2	101.5	129.4
	Total	10394.7	10338.4	374.5	14425.1	1047.3	138.3	36756.7

### Spatial distribution of ecological services in Zhangjiakou

#### Water and soil conservation

**Figure 2:**
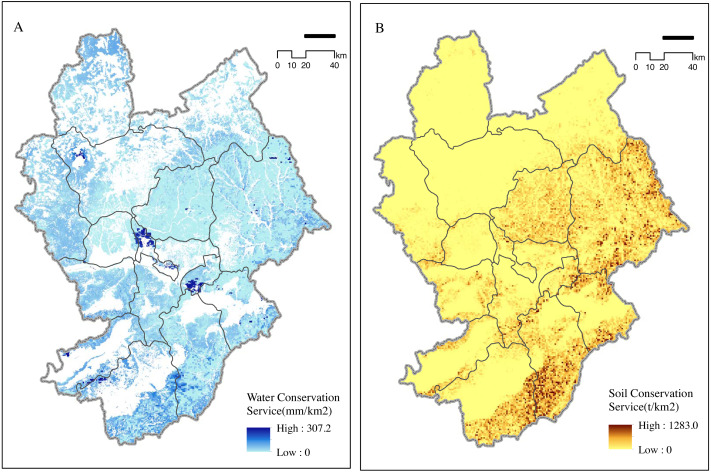
Importance of (A) water and (B) soil conservation. Zhangjiakou, an important windbreak and sand stabilization barrier in Beijing Tianjin Hebei area, is of great importance in sand fixation.

Water conservation areas in Zhangjiakou were widely distributed, especially throughout the southern Zhangjiakou Nature Reserves, with an area of 307 km^2^. The areas along the Sanggan River and Yanghe River Valley were highly important for water conservation and contributed to the regulation of the water cycle and prevention of siltation in rivers, lakes, and reservoirs. Weak areas of water conservation were detected south of Zhangjiakou (Fig. 2A). Water conservation areas with extreme functional importance accounted for 1.8% of the area, those with moderate importance accounted for 2.08%, and those of general functional importance made up the largest proportion (Table 2).

**Table 2:** Water conservation and soil conservation classification statistics in 2010.

Function Level	Water Conservation		Soil Conservation	
	Acreage (km^2^)	Area ratio (%)	Acreage (km^2^)	Area ratio (%)
Very Important	661.96	1.80	1828.07	4.97
Important	763.35	2.08	4921.71	13.39
Moderate	3479.34	9.47	8749.80	23.80
Common	31851.08	86.66	21259.12	57.83

Soil conservation areas of Zhangjiakou were mainly distributed in the eastern and southern counties. The areas with weak soil conservation function were distributed in the northwestern and southwestern counties (Fig. 2B). Distribution of soil conservation function was related to the geomorphic units of Zhangjiakou. The northern part of Zhangjiakou is at the southern edge of the Inner Mongolia Plateau and has the shape of a wavy plateau. Soil conservation function in this area was not evident. The southern and eastern areas, with medium-sized basins, rolling mountains, and ravines had important soil conservation functions. The total area of the soil conservation function in Zhangjiakou was 21,259.12 km^2^, all of which were of general functional importance. Very important areas and areas for water and soil conservation in the city accounted for about 17.4% of the total area, and were distributed among a variety of conservation areas in Zhangjiakou (Table 2).

#### Sandstorm prevention and biodiversity maintenance

**Figure 3:**
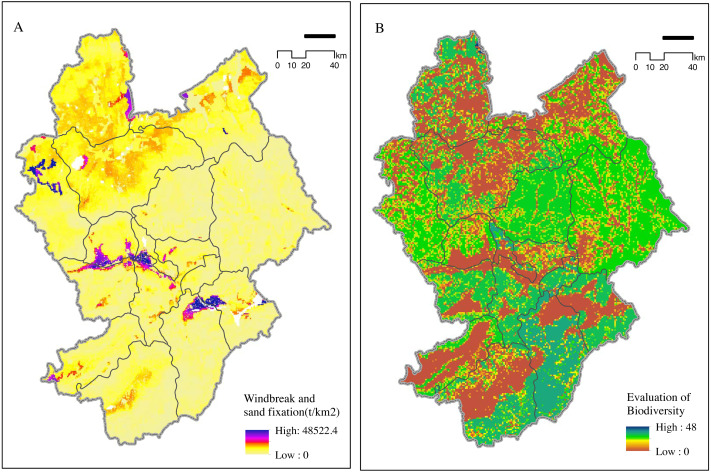
Importance of (A) windbreak and (B) biodiversity maintenance. The increase in construction land coincided with the decrease in cultivated land mostly at edges of artificial surfaces.

Zhangjiakou is an important sandstorm prevention barrier in the Beijing Tianjin Hebei area. Its sandstorm prevention function was most important in western Zhangjiakou (Fig. 3A), while areas with a weak sandstorm prevention function were distributed in the east and south of Zhangjiakou. Zhangjiakou district exhibited highest importance for sandstorm prevention with a total area of about 1,992.5 km^2^ (Table 3). The land deemed most important and important accounted for 15.63% of the total area. In addition, the proportion of moderately important areas was relatively high, accounting for 51.16%.

**Table 3:** Sand fixation and biodiversity function classification statistics in 2010. The proportion of extremely important areas in the total area was about 6.57form of patches within the nature reserve.

Function Level	Sand Fixation		Biodiversity	
	Acreage (km^2^)	Area ratio (%)	Acreage (km^2^)	Area ratio (%)
Very Important	1992.50	5.42	2417.26	6.57
Important	3753.95	10.21	1139.27	3.09
Moderate	18804.50	51.16	5208.13	14.17
Common	12205.75	33.21	27992.04	76.15

Areas with a functional importance to biodiversity in Zhangjiakou were mainly distributed in the central and southern areas. The most important functional areas were found mostly in the nature reserves in southwestern China, totaling 2,417.26 km^2^ (Fig. 3B). Proportion of extremely important areas (of total area) was about 6.57%, mainly in the form of patches within the nature reserve (Table 3). Proportion of moderately important areas was about 14.17% of the total area. Functional areas are very important for protecting and maintaining biodiversity.

### Impact of artificial surface expansion on regional ecological service

Calculation of land-use demand for Zhangjiakou from 2005 to 2015 showed that natural protection was generally important, water bodies and forest lands were strictly protected, and conversion rate of farmland and forest land to construction land decreased. We created a new transfer probability matrix and simulated land use demand in 2025 using the Markov model.

Simulation results showed that ROC values for different land use types were ≥ 0.76, indicating that the selected driving factors were important for many land-use types. The maximum ROC value of forest land was 0.87, indicating that the selected driving factors best illustrated forest land. The ROC value of artificial surface, grassland, and farmland was >0.8, and that of other land types was lower.

The kappa coefficient was 0.84. A comparison of simulation results for 2015 with the current land distribution in Zhangjiakou showed that the CLUE-S model accurately predicted land types in the study area. We expected to optimize the spatial development pattern of land, promote the improvement of the environment, and ensure the ecological security barrier of Beijing, Tianjin, and Hebei by combining the strategic objectives of Zhangjiakou. Natural protection was given priority in the prediction of land use demand in Zhangjiakou in 2025.

**Figure 4:**
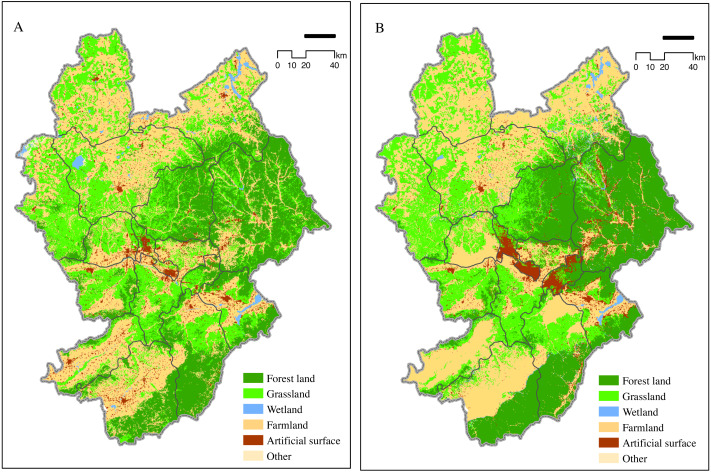
Zhangjiakou 2015 land use map (A) and 2025 simulation map (B). The calculation for the land-use demand for Zhangjiakou from 2005 to 2015 showed that natural protection was generally important.

Changes in land use in Zhangjiakou City in 2025 were mainly reflected in the increase in artificial surface, particularly in the conversion of farmland to artificial surface (Fig. 4). Forest land and grassland were converted to artificial surface at the country level. In addition, some grassland has also been converted into forest land due to the recent implementation of large-scale ecological restoration projects. New artificial surface area increased by 226.1 km^2^ in 2025, and the decrease in farmland and the increase in ecological land occurred mostly in the Bashang Plateau (Table 4). The increase in construction land coincided with the decrease in cultivated land, particularly at the edges of artificial surfaces, indicating that further increases in artificial surface area will involve declines in farmland areas.

**Table 4:** The area occupied by Zhang Jiakou urban expansion for integrated ecosystem services in 2025. large-scale ecological restoration projects, some grassland has also been converted into forest land. New artificial surface area increased by 226.1 km^2^ in 2025.

Invaded Eco-service function	Type	Acreage/km^2^	Total acreage
Moderate	Water Conservation	6.52	20.58
	Soil Conservation	4.57	
	Sand Fixation	4.08	
	Biodiversity	5.41	
Important	Biodiversity +Sand Fixation	1.11	6.37
	Biodiversity+Soil Conservation	1.34	
	Biodiversity +Water Conservation	1.27	
	Sand Fixation+Water Conservation	1.41	
	Soil Conservation+Water Conservation	1.24	
Very Important	Biodiversity +Sand Fixation+Water Conservation	3.12	4.16
	Biodiversity +Soil Conservation+Water Conservation	2.23	

Land-use change from 2015 to 2025 was estimated to involve urban expansion into areas with ecological service function of moderate level of importance or above. The area with a moderate importance service function accounted for 20.58 km^2^. Important service function areas totaled 6.37 km^2^ and were mostly found in municipal districts. Based on our results, we predict that ecological services, including windbreak, sand stabilization, and water conservation in Zhangjiakou may decrease in 2025. Considering the impact of predicted city expansion on ecological service function by 2025, municipal districts of Southern Zhangjiakou retained moderately important ecological service function, with a total of 15.72 km^2^ , while 50.53% of the total were damaged areas (Table 4). The impact on ecological services will have a negative effect on ecological security around Zhangjiakou. Therefore, Zhangjiakou should focus on protecting the established forest vegetation in the south in future land planning.

## Discussion

### Effects of land use changes on ecological services

Studies have shown that a decrease in farm and forest land is a driving factor of regional development. Agricultural production is impacted by global change and the frequent occurrence of extreme weather events result in losses (Cao et al., 2010). Our main objective was to quantify temporal and spatial changes in ecological service functions. Our early research in Zhangjiakou provided new evidence for the decreasing ecological service function caused by land use change (Xiao & Xiao, 2018). In this study, we found clear evidence that water retention capacity in Zhangjiakou fluctuated from 2000 to 2015, with a decreasing trend. A decline in water retention capacity was found in almost the entire western region (Carbone, 2014), showing that land use change and urban expansion led to a decline in ecological quality.

The urban ecosystem in Zhangjiakou is characterized by a rapid increase in artificial surface, and disappearance of large areas of farmland and key areas. The plateau climate of Zhangjiakou also has strong regional differences and distinct dry and wet seasons, which may increase the risk of decline of ecological service function in the area. Our research focused on the ecological barrier area of Beijing-Tianjin and Hebei, which is very important for ecological security of the capital of China. The eco-environmental effects of changes in land use are sensitive to the natural services of the ecosystems in the Beijing Tianjin Hebei region (Shrestha et al., 2015).

Recent studies on the impacts of changes in land use on ecological service functions in northern China showed that the average temperature in Beijing, Tianjin, and Hebei increased by 0.22 °C between 1960 and 2010 as a result of global climate change, and that these effects are increasing (Ouyang et al., 2016). Land use in Zhangjiakou has changed significantly since the 1980s, and the intensity of land disturbance to ecosystem services has increased since that time (Gong et al., 2012). We provided empirical evidence that, in the future, land use change will be characterized by an increase in urban areas, and the conversion from forest and cultivated lands to urban land. Other likely conversions will involve the development of forests and grasslands to artificial surfaces (Carmen et al., 2018). We found that the decrease in cultivated and forest land mainly occurred in valley areas downstream of dams, and changes to land use areas in Zhangjiakou were undertaken to create areas suitable for human habitation (Wu et al., 2017). Topography has a direct impact on the increase and decrease of ecosystem services and limits changes in land use. The change in land use may be the most direct reason for the change in ecological service functions in Zhangjiakou. The ecological environment effect directly determines ecological security of Beijing-Tianjin and Hebei regions, regardless of the reason for the changes in land use areas.

### Relationship between ecological service functions

Forests provide a wide range of ecological service functions, including improvement in soil conditions (Chettri et al., 2021). However, not all forests exhibit the same functions. Thus, it is particularly important to enhance ecosystem services in sensitive areas in the eastern and southern areas of Zhangjiakou. The balance between natural and human-made ecosystems will be reduced without the identification and protection of important ecological service functions (Xiao & Xiao, 2019). We found that the identification and restoration of ecological services in Zhangjiakou will significantly increase ecological functions in the area, thereby reducing ecological risks in Beijing, Tianjin, and Hebei. The soil conservation function of Zhangjiakou is mainly distributed in the eastern and southern areas and areas with low soil conservation functions are mainly distributed in the northwest and southwest. The different levels of soil conservation may be related to the spatial distribution characteristics and geomorphic units of Zhangjiakou (Sylla et al., 2021).

We found that there was a spatial overlap between water conservation and sand stabilization, and these results were reflected in a previous study (Cao et al., 2016). The spatial overlap of windbreak, sand fixation, and water conservation indicated that common features existed among these ecological services. Areas with high overlap and spatial consistency were located near Zhangjiakou City and the Sanggan River, and involved intensive human activities and frequent land use changes. The most important area of windbreak and sand fixation was located in Zhangjiakou City, where it accounted for 15.63% of the total area. The proportion of areas with moderately important service functions was relatively high, accounting for 51.16% of the area. It is evident that windbreak and sand fixation services in Zhangjiakou are already strained, and continued high-intensity human activities are likely to exceed regional ecosystem capacity (De Souza Dupas & Silva, 2021).

### The importance of an ecological barrier

Biodiversity embodies a complex interrelationship between organisms and the environment. Protecting regional biodiversity will ensure that ecosystem services remain in place (Sun, Miao & Duan, 2017). Areas with important levels of water conservation are widely distributed in Zhangjiakou, especially in the nature reserves of southern Zhangjiakou. The most important area of water conservation surrounds the Sanggan River and Yanghe River Valley, and is important for regulating the water cycle and preventing siltation in rivers, lakes, and reservoirs (Gong et al., 2016). This indicates that full consideration should be given to the allocation of agricultural land and water areas when planning for land use. Further research will elucidate effects on environmental health. Policy makers should also consider the loss of ecological service values caused by land use change when considering the social and economic benefits of land use, and a dynamic balance should be determined between the two (Flood, Mahon & McDonagh, 2021).

Ecosystems are complex and not fully understood, and attempts to restore ecosystems should be strictly verified to prepare for unexpected consequences before humans intervene in ecosystem stability (Zhao et al., 2021)The effectiveness of restoration measures in the provision of biodiversity and ecosystem services has not been systematically assessed. Our study showed that the biodiversity function in Zhangjiakou is mainly distributed in the central and southern areas. The most important functional areas are distributed mostly in the nature reserves in southwestern China with an area of approximately 2,417.26 km^2^. Our results quantified the importance of land use changes to ecological service function (Wu et al., 2021). The change of grassland, water area, and forest land is the main factor affecting ecological service functions. Changes to the woodlands and grasslands should be strictly monitored in important ecological function areas. Comprehensive management of cultivated and forest lands should be improved, and returning farmland to forest and grassland should be undertaken in a planned way to improve the quality of ecological environment (Chen et al., 2020).

### Implications of results

We identified and quantified the impact of land use change on the importance of ecosystem services. Protection of these small areas will help Zhangjiakou protect key areas of biodiversity. We found that the current nature reserve did not maximize the provision of ecosystem service for windbreak, sand stabilization, and water conservation. The degradation of the ecosystem will aggravate the shortage of water resources around Zhangjiakou and increase the occurrence of sandstorms, with a negative feedback on ecological security around Zhangjiakou. Therefore, future land planning for Zhangjiakou should focus on protecting forest vegetation resources in the south (Gou et al., 2021).

Policymakers contemplating financial incentives to increase the flow of sustainable ecosystem services should consider what incentives may effectively achieve policy objectives (Xiao, Xiao & Xiong Q. Yang, 2020). Effective policies and programs must be scientifically appropriate, and require long-term support from those affected by the policies. Few interventions have encouraged local residents to reduce their habitat use even when a conservation project affects the local resource utilization strategy over a short period of time (Zoysa, 2021). For example, paying to plant trees will encourage afforestation as long as the payment continues, but when payments stop, residents return to old habits to use trees for an unsustainable supply of fuel wood. By contrast, other projects, which encourage the cultivation of fruit trees, will provide greater benefits because fruit can be used for food and for revenue generated by sales (Gutierrez-Garzon et al., 2021).

## Conclusions

In this study, we adopted and applied an ecological functional importance evaluation for Zhangjiakou City. We identified areas of importance for ecological functions including water conservation, soil conservation, sandstorm prevention, and biodiversity. We found that the CLUE-S model can simulate the dynamic change of urban development in Zhangjiakou based on land use in Zhangjiakou in 2005, 2010, and 2015. Predicting changes in Zhangjiakou’s land use can help stakeholders understand ecosystem service conservation and maintenance and support environmental protection; stakeholders can make informed decisions that promote a harmonious development of economy and the environment.

Afforestation in Zhangjiakou should be strictly controlled to alleviate the declining groundwater levels and attention should be paid to ecosystem restoration to slow the effects of climate change. It is necessary to clarify goals for ecological protection in view of respecting nature, conforming to nature, and protecting nature, and to adhere to environmental protection and natural recovery. It is important to implement ecological protection and restoration projects and to comprehensively improve the health, stability, and service function of ecosystems. A strategy for sustainable environmental remediation must clearly involve ecological, economic, and social development. In order to effectively reduce the cost of ecosystem restoration and achieve sustainable development, relevant administrative bodies must make full use of natural recovery to restore sustainable natural ecosystems under local conditions.

## Supplemental Information

10.7717/peerj.12122/supp-1 Supplemental Information 1 Transfer between Zhangjiakou Ecosystem Types (2000-2015) Between 2000 and 2015, Zhangjiakou City has experienced a rapid increase in artificial surface area

10.7717/peerj.12122/supp-2 Supplemental Information 2 2010 Soil and Water Conservation Classification Statistics. It can be opened with geographic information system The total area of soil and water conservation in Zhangjiakou City is 21259.12 km^2^, which has universal functional significance

10.7717/peerj.12122/supp-3 Supplemental Information 3 Spatial Distribution of Water Conservation and Soil Conservation Service The soil and water conservation functions of Zhangjiakou are mainly distributed in the east and south. It can be opened with geographic information system

10.7717/peerj.12122/supp-4 Supplemental Information 4 Importance of Windbreak and Biodiversity maintenance The functional importance of biodiversity in Zhangjiakou was mainly distributed in its central and southern areas.

10.7717/peerj.12122/supp-5 Supplemental Information 5 Zhangjiakou 2015 land use map and 2025 simulation map Simulate the process data of land use change in 2025

## Additional Information and Declarations

### Data Availability

The following information was supplied regarding data availability: Raw data are available in the Supplemental Files. Use geographic information system software (ArcGIS). Specific steps: 1. Open the ArcGIS login interface. 2. Select the ”Add” button, find the folder where the data is located, and click the data to be loaded to load. 3. The data is displayed.
